# Comparisons of 3-Dimensional Conformal and Intensity-Modulated Neutron Therapy for Head and Neck Cancers

**DOI:** 10.14338/IJPT-20-00059.1

**Published:** 2021-09-14

**Authors:** Natalie Viscariello, Matthew D. Greer, Upendra Parvathaneni, Jay J. Liao, George E. Laramore, Robert D. Stewart

**Affiliations:** Department of Radiation Oncology, University of Washington, Seattle, WA, USA

**Keywords:** fast neutron therapy, treatment planning

## Abstract

**Purpose:**

Neutron therapy is a high linear energy transfer modality that is useful for the treatment of radioresistant head and neck (H&N) cancers. It has been limited to 3-dimensioanal conformal-based fast-neutron therapy (3DCNT), but recent technical advances have enabled the clinical implementation of intensity-modulated neutron therapy (IMNT). This study evaluated the comparative dosimetry of IMNT and 3DCNT plans for the treatment of H&N cancers.

**Materials and Methods:**

Seven H&N IMNT plans were retrospectively created for patients previously treated with 3DCNT at the University of Washington (Seattle). A custom RayStation model with neutron-specific scattering kernels was used for inverse planning. Organ-at-risk (OAR) objectives from the original 3DCNT plan were initially used and were then systematically reduced to investigate the feasibility of improving a therapeutic ratio, defined as the ratio of the mean tumor to OAR dose. The IMNT and 3DCNT plan quality was evaluated using the therapeutic ratio, isodose contours, and dose volume histograms.

**Results:**

When compared with the 3DCNT plans, IMNT reduces the OAR dose for the equivalent tumor coverage. Moreover, IMNT is most advantageous for OARs in close spatial proximity to the target. For the 7 patients with H&N cancers examined, the therapeutic ratio for IMNT increased by an average of 56% when compared with the 3DCNT. The maximum OAR dose was reduced by an average of 20.5% and 20.7% for the spinal cord and temporal lobe, respectively. The mean dose to the larynx decreased by an average of 80%.

**Conclusion:**

The IMNT significantly decreases the OAR doses compared with 3DCNT and provides comparable tumor coverage. Improvements in the therapeutic ratio with IMNT are especially significant for dose-limiting OARs near tumor targets. Moreover, IMNT provides superior sparing of healthy tissues and creates significant new opportunities to improve the care of patients with H&N cancers treated with neutron therapy.

## Introduction

High-energy (fast) neutron therapy is an effective treatment for radiation-resistant tumors and has been used in the management of many sites, including salivary gland tumors, locally advanced prostate cancer, melanoma, soft tissue sarcomas, and recurrent cancers [1–6]. Fast neutron therapy is considered a high linear energy transfer (LET) treatment modality. Neutrons interact with target nuclei to produce secondary charged particles, such as protons, alpha particles, and more-massive (*Z* > 2) ions. Neutrons also creates high-energy gamma rays through (*n*, γ) reactions, primarily through interactions with hydrogen. In the University of Washington (UW, Seattle) Clinical Neutron Therapy System (CNTS), approximately 2% to 10% of the absorbed dose is from gamma rays; up to 85% is from recoil protons (average kinetic energy, approximately 14-18 MeV), and the remaining arises from alpha particles (average kinetic energy, approximately 4-6 MeV) and *Z* > 2 ions [7]. The relative contribution of these particles varies with field size, depth, distance from the central axis, and tissue composition. A 15-MeV proton has an LET of approximately 3.2 kilo electron volts per micrometer keV/μm and a range in water of 2.6 mm (diameter, approximately 260 human cells), and a 5-MeV alpha particle has an LET of approximately 90 keV/μm and a range of 37.4 μm. Lower-energy protons and alpha particles can have an LET as high as 78 keV/μm and 228 keV/μm, respectively. For comparison, the megavoltage (MV) x-rays widely used for most patients with head and neck treatments have an LET of < 0.3 keV/μm (100-1000 times smaller than the LET of the charged particles produced in fast-neutron therapy).

This difference in LET produced by indirectly ionizing neutrons and x-rays creates unique spatial patterns of DNA lesions within 1 or 2 turns of the DNA (10-20 base pair [bp]) as well as differences in the overall number of clusters of DNA lesions separated by 10s or 100s of nanometers [8, 9]. Compared with MV x-rays and other low-LET radiation, the densely ionizing (high-LET) charged particles create large amounts of challenging-to-repair DNA damage, including DNA double-strand breaks (DSBs) and other complex clusters of DNA lesions. The complex, non-DSB clusters created by high-LET radiation also include challenging-to-repair single-strand breaks with additional base damage or abasic sites within a few base pairs, as well as clusters of DNA lesions composed solely of abasic sites or base damage (ie, the cluster does not contain any strand breaks). However, DSBs are usually considered the most critical form of sublethal and potentially lethal damage because lethal chromosome aberrations are largely formed through the incorrect rejoining of pairs of DSBs [10, 11]. The CNTS fast neutrons create 2.7 times as many DSBs per unit of absorbed dose as γ rays from cobalt 60 (^60^Co) or high-energy x-rays [9]. High-LET radiation is also effective at overcoming multiple mechanisms of radiation resistance (eg, hypoxia) and can be as much as 2 to 6 times more likely to kill a cell per unit of absorbed dose than MV x-rays [12, 13]. Fast neutrons have been found to be effective at achieving local control of radioresistant tumors [3, 14, 15]. The increase in biologic damage per unit dose for molecular, cellular, and clinical endpoints is often quantified in terms of a radiation's relative biological effectiveness (RBE). That is, fast-neutron RBE is 2.7 for the endpoint of DSB induction and approximately 3 to 6 for the endpoint of reproductive cell survival using the repair-misrepair-fixation cell-survival model and representative parameters for human cells [13].The UW CNTS was the first hospital-based facility in the United States with a gantry, wedges, and multileaf collimators (MLCs) capable of delivering 3-dimensional (3D) conformal neutron therapy [16]. Since October 1984, we have treated > 3200 patients with fast-neutron therapy. Clinical experience with fast-neutron therapy at the UW suggests that the RBE for cell survival (approximately 3-6) is similar to the RBE for local tumor control and for dose-limiting organs at risk (OARs). For patients with head and neck cancers, OAR constraints in use at the UW (optic nerve, lens of the eye, brainstem, among others) correspond to a neutron RBE of 2.9 to 6.2 [17]. The tissue-specific RBE mainly depends on the clinical endpoint of interest, tissue α/β, and dose per fraction. In general, neutron RBE tends to increase as the dose per fraction decreases and as the tissue α/β decreases. For tumor targets, the total prescription dose for a fast-neutron treatment is usually a factor of 3 to 5 less than the total doses used for MV x-ray treatments delivered at 2 Gy/d, which corresponds to a tumor RBE of about 3 to 5. For example, MV x-ray doses on the order of 60 to 70 Gy are often used for head and neck cancers [18, 19] compared with total neutron doses of approximately 18 Gy delivered as 1.15 Gy/d (RBE, approximately 3.3-3.9). Of note, the RBE for fast-neutron therapy (approximately 3-6) is quite similar to the RBE for therapeutic carbon ions [13, 20].

The large RBE for OAR toxicity, especially for late-responding tissues, often becomes dose limiting for some treatment sites and, ultimately, constrains the use of 3D conformal neutron therapy (3DCNT) or necessitates additional radiation with higher conformality (although with lower LET), such as stereotactic techniques or particle therapy. In our practice, Gamma Knife or protons are used, depending on the clinical scenario [21–23]. The OAR toxicity is of special concern in the treatment of salivary gland tumors of the head and neck, the most-common, contemporary indication for neutron therapy at the UW. Many salivary gland tumors demonstrate neurotropism and invasion into the base of the skull, requiring lower tumor prescriptions to limit neutron dose to the brain and/or spinal cord [24]. For example, to avoid central nervous system complications, the maximum neutron dose to the temporal lobe of the brain is limited to 12.5 to 13.5 Gy (approximately 66% of the desired tumor dose in 1.15 Gy daily fractions), which translates to approximately 50 to 60 Gy MV x-ray equivalent in 2 Gy daily fractions (RBE approximately 3.7-4.8). This creates a limitation with intentional underdosage of part of the target volume close to the skull base, especially for adenoid cystic carcinomas of either major or minor salivary gland origin with perineural tumor spread along the facial or trigeminal nerve pathways. Late neutron toxicity appears to be a function of absolute dose more than a function of daily fraction size [25, 26], and many locally advanced salivary gland tumors require compromises in tumor coverage or supplementation with other modalities to meet tumor-coverage goals.

One approach to reducing treatment toxicity is to limit the physical dose delivered to relevant OARs, especially those OARs in close spatial proximity to a tumor target. This can be accomplished with technologies such as MLCs wedges, multiple beam angles, and advanced planning techniques that create highly modulated dose distributions (ie, intensity-modulated radiation therapy [IMRT]) through inverse planning. In prospective clinical studies, IMRT has been shown to decrease the OAR toxicity from photon radiation when compared with 3DCNT [27–31]. Intensity-modulated neutron therapy (IMNT) has been previously commissioned [32] and has shown dosimetric advantages, although planning studies have been limited to the prostate [4, 33]. The effect of IMNT on OAR doses in patients with head and neck cancers has yet to be evaluated, to our knowledge, which is the focus of this work.

In addition to dosimetric benefits, the use of dynamic MLCs in IMNT also offers the practical advantage of reduced treatment time because the same or higher levels of dose modulation can be achieved without the need for wedge fields (field setup times are longer for wedged fields than they are for open fields). A sequence of segmented fields can also be automatically delivered as a single IMNT field, whereas therapists must enter the treatment vault each time a new 3DCNT field is set up. Despite its advantages, implementation of IMNT has been limited, largely because of engineering and treatment planning system constraints. Recently, the UW updated to the leaf collimator system in the CNTS treatment head to enable the rapid delivery of IMNT fields, and we have also created a treatment planning machine model capable of inverse planning with neutron-specific scattering kernels [34, 35]. This change from forward-planned 3DCNT delivery to highly modulated IMNT planning creates new opportunities to reduce OAR toxicity in patients with head and neck cancers. The ability to reduce neutron dose through the use of IMNT is especially important in patients with prior radiation therapy because dose-limiting OARs may not have fully recovered between treatment courses. The goal of this work was to evaluate the potential dosimetric advantages of IMNT for head and neck cancers through retrospective replanning of patients previously treated with 3DCNT.

### Materials and Methods

The physical and dosimetric characteristics of the CNTS system have previously been described in detail [7, 36]. In brief, the CNTS generates fast neutrons by directing 50.5 MeV ^1^H^+^ (positively charged ion of a hydrogen atom) ions onto a 10.5-mm-thick beryllium (Be) target with copper backing. Fast neutrons generated in the Be target and copper backing pass through a primary collimator, a flattening filter, a monitor unit chamber, an optional (30°, 45°, or 60°) wedge assembly, and a specialized, neutron-specific MLC, which comprises 40 individually moveable leaves; the leaves can be positioned with an accuracy of ± 2 mm at the isocenter. The treatment head is mounted on a gantry capable of ± 184° of rotation around the radiation isocenter located at a source to axis distance of 150 cm. The combined MLC and wedge assembly can independently rotate (76°-283°) about the coincident MLC mechanical rotation axis and radiation beam central axis. Overall, CNTS neutrons have depth-dose characteristics similar to 6-MV x-rays, with a depth of maximum dose approximately 1.7 cm and a 90%/20% field penumbra within approximately 10 mm [7].

For the past 35 years, 3DCNT has been the standard of care for patients treated with the CNTS. With the transition from Pinnacle (Phillips Radiation Oncology Systems, Fitchburg, Wisconsin) [37] to a custom build of the RayStation (Raysearch Laboratories, Stockholm, Sweden) treatment planning system with neutron-specific scattering kernels [7, 35], we now have the ability to perform inverse planning. These treatment planning advances have allowed us to take advantage of IMNT.

A retrospective, institutional review board–approved evaluation of 7 patients with head and neck cancers who were previously planned with 3DCNT was conducted. The head and neck anatomic sites investigated were 3 (43%) parotid and 4 (57%) oral cavity minor salivary gland tumors treated with Raystation-generated plans between June 2020 and August 2020. This allowed for a direct comparison between 3DCNT and IMNT using the same scattering kernels and beam model in the Raystation. **Supplemental Table** lists the dose fractionation schemes for the patients as well as OAR constraints that were used in the clinical plans. For conventional 3DCNT at our center, the treatment field is typically defined with a 1-cm margin around the planning target volume (PTV). The number and gantry angle of the beams are chosen by the certified medical dosimetrist on a case-by-case basis. Typical head and neck plans consist of ≥ 3 coplanar beams with wedges of 30°, 45°, or 60°.

The IMNT model allows for inverse planning with optimization objectives and several segments per beam angle. Structures from the original 3DCNT plan, including the PTV and OARs, were not modified for the IMNT planning. Clinical goals for relevant OARs and the PTV from the clinically approved 3DCNT plans were initially used as input objectives in the optimization process. The PTV uniformity was maintained by creating a maximum dose constraint of 110% of the prescription, and 100% of the volume was set to receive ≥ 95% of the prescription dose. Both the 3DCNT and IMNT plans were prescribed to the same dose calculation point to maintain consistency in normalization. A maximum of 16 segments per field was allowed in the optimization process. Optimization weights for OARs were iteratively adjusted until the clinical goals of the plan were met. To evaluate the ability of inverse planning to reduce OAR doses to less than those commonly achievable with 3DCNT, the numerical value of the OAR clinical constraints was reduced in the optimizer by 10% and 20%; henceforth, they are referred to as IMNT_10_ and IMNT_20_, respectively.

After the IMNT plans were created, dose-volume histogram (DVH) parameters were used to evaluate and compare the plans. For the PTV, the mean dose, D_95%_ (minimum dose that covers 95% of the PTV volume), D_99%_ (near minimum dose), and D_2%_ (near maximum dose) were considered. For OARs, the mean dose and D_2%_ were calculated. From these DVH metrics, a therapeutic ratio (TR) was calculated for each OAR as follows:

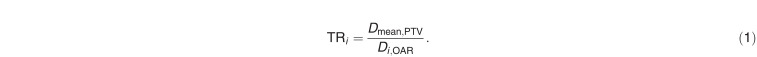



The subscript *i* refers to the maximum or mean, depending on the type of OAR objective. The TR is a useful metric because the investigated plans had a large range of prescription doses (450-2000 cGy), and a dimensionless metric of the relative plan quality is useful for the comparison of the 2 planning methods. Differences in 3DCNT and IMNT plan quality for the target volumes and OARs were compared with nonparametric Wilcoxon signed-rank tests. Plan quality was also assessed through direct comparisons of standard DVHs and the therapeutic ratios.

## Results

**Figure 1.**
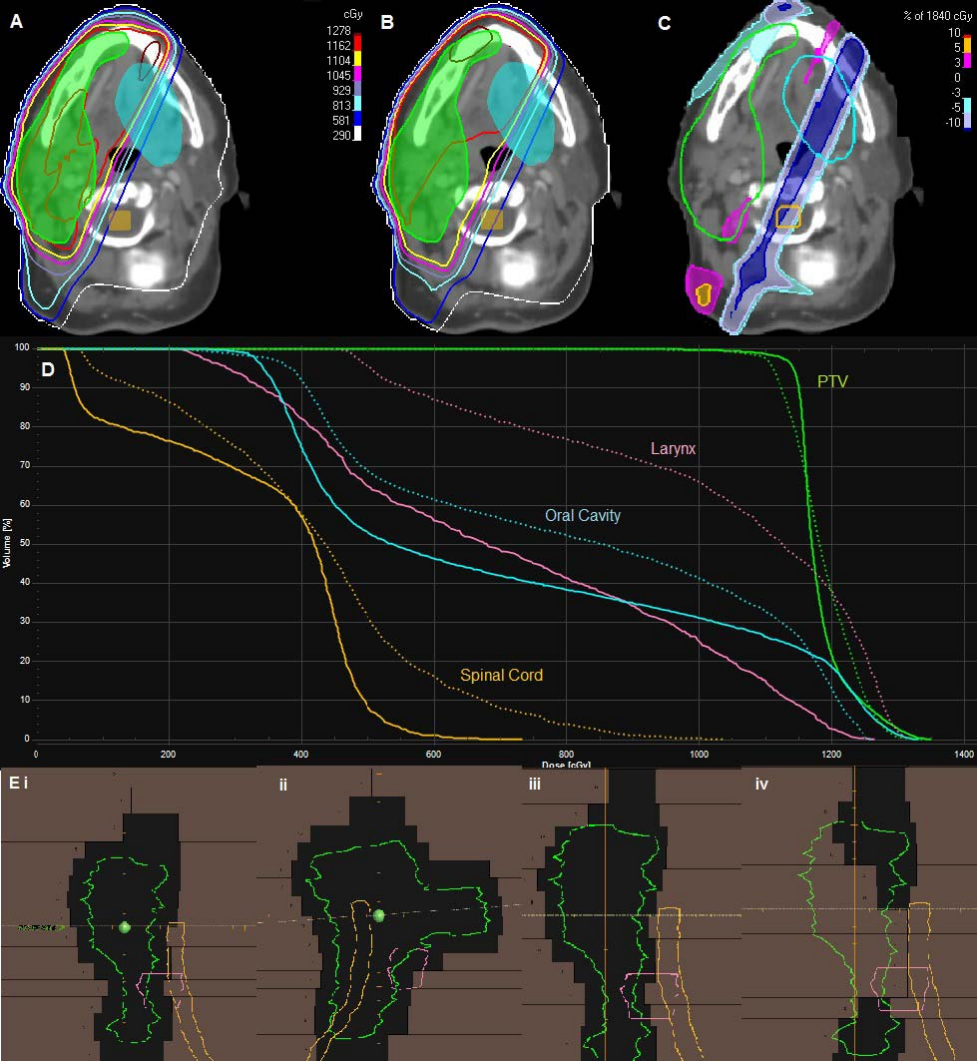
Representative case of 1 patient with adenoid cystic carinoma of the parotid. (A) The intensity-modulated neutron therapy (IMNT) plan. (B) The clinically approved 3-dimensional conformal neutron therapy (3DCNT) plan. For both, the planning target volume (PTV) is a filled contour (green), and the oral cavity (blue) and spinal cord (yellow) are shown. (C) Dose-difference comparison (IMNT-3DCNT) in the same axial slice, where the filled dark blue area indicates a reduction in the dose for the IMNT plan. (D) Dose-volume histogram of the 2 plans. Solid lines indicate IMNT, and dashed are 3DCNT. (E) Beam-eye views for the 3DCNT plan (i-ii), IMNT plan (ii-iv), and from the same beam angle, but representing differing segments in the IMNT plan (iii and iv) .

**Figure 1** shows a comparison of the DVHs for representative 3DCNT (dotted lines) and IMNT (solid lines) plans for a patient with adenoid cystic carcinoma of the right parotid. Isodose distributions are overlaid on the planning computed tomography, with a 2D dose difference shown in **Figure 1C**. For clarity, only the larynx, oral cavity, and spinal cord OARs are shown on the DVH and the computed tomography. The OARs that were present in most plans are tabulated in **Table 1**. The initial 3DCNT plan in **Figure 1B** consisted of 2 wedged and 1 en face field, which leads to greater dose at the thinner portion, or “toe,” of the wedge. The IMNT plan shows a substantial reduction of dose in the previously wedged portions of the fields (**Figure 1C**).

**Table 1.** Dose-volume histogram (DVH) metrics for the 7 patient plans in this study. Relevant organs at risk (OARs) for each plan are listed; absence of an OAR indicates that it was not contoured in the clinical plan. All values are expressed in centigray (cGy), unless otherwise noted as percentages.

**Patient No. and treatment**	**PTV statistics**				**OAR statistics, cGy**					
	**Mean**	**D_95_, %**	**D_99_, %**	**D_2_, %**	**Spinal cord maximum**	**Temporal lobe maximum**	**Cerebellum maximum**	**Cochlea maximum**	**Larynx mean**	**Oral cavity mean**
Patient 1										
3DCNT	1185	96.6	92.5	111.8	861	1225	—	—	1024	813
IMNT	1173	99.4	94.3	113.7	559	1102	—	—	710	711
IMNT_10_	1153	89.3	77.0	109.9	565	1009	—	—	619	548
IMNT_20_	1152	94.2	77.2	114.6	539	929	—	—	542	484
Patient 2										
3DCNT	1151	93.0	84.3	106.6	557	1068	676	179	221	542
IMNT	1175	94.0	87.2	107.0	438	1027	847	188	178	288
IMNT_10_	1134	85.9	72.5	111.0	338	940	805	150	146	264
IMNT_20_	1125	82.0	60.4	112.9	295	788	584	125	140	226
Patient 3										
3DCNT	483	102.7	100.9	116.4	148	152	129	214	220	—
IMNT	488	100.2	95.3	120.7	103	150	118	219	139	—
IMNT_10_	494	100.2	94.7	121.3	96	150	121	223	122	—
IMNT_20_	495	100.4	92.0	121.6	94	147	107	180	112	—
Patient 4										
3DCNT	452	95.3	80.7	107.3	79	69	103	178	192	—
IMNT	460	95.3	81.6	112.4	76	38	48	55	138	—
IMNT_10_	462	94.4	82.0	115.6	70	39	48	56	127	—
IMNT_20_	465	94.0	81.8	112.9	64	39	48	48	115	—
Patient 5										
3DCNT	458	97.6	94.4	107.6	50	68	43	55	—	—
IMNT	467	98.7	94.7	111.3	44	53	37	47	—	—
IMNT_10_	468	97.8	94.4	113.6	44	53	37	47	—	—
IMNT_20_	467	96.9	93.3	118.2	44	53	37	47	—	—
Patient 6										
3DCNT	1141	95.5	93.0	104.2	734	1157	—	—	—	—
IMNT	1158	95.5	92.4	105.5	749	1122	—	—	—	—
IMNT_10_	1134	92.4	88.4	103.9	740	1108	—	—	—	—
IMNT_20_	1136	91.4	87.7	102.8	714	1000	—	—	—	—
Patient 7										
3DCNT	2038	94.8	91.5	107.7	297	1129	1213	—	—	—
IMNT	1991	94.4	86.5	103.8	317	974	996	—	—	—
IMNT_10_	1995	92.1	81.5	102.6	295	956	900	—	—	—
IMNT_20_	1989	92.5	79.1	105.2	271	988	830	—	—	—

**Abbreviations:** PTV, planning target volume; D_95_, minimum dose that covers 95% of the PTV volume; D_99%_, near the minimum dose; D_2%_, near the maximum dose; 3DCNT, 3-dimensional conformal neutron therapy; IMNT, intensity-modulated neutron therapy; IMNT_10_, reductions from the initial OAR constraints in the optimization by 10%; IMNT_20_, reductions from the initial OAR constraints in the optimization by 20%.

The DVH statistics are presented in **Table 1** as a function of planning technique. The PTV coverage is acceptable for 18 of 21 of the IMNT plans; D_95%_ was kept to ≥ 90% of the prescribed dose. The D_95%_ was slightly greater for the 3DCNT in 5 plans, indicating a slightly more-pronounced “shoulder” in the DVH curve. The IMNT tended to have higher near-maximum dose values when compared with the 3DCNT. **Table 2** compares 3DCNT and IMNT average plan metrics with OAR constraints reduced by 10% (IMNT_10_) and 20% (IMNT_20_), respectively. For IMNT plans optimized with the same OAR constraints as the 3DCNT plan, the PTV coverage and the mean and maximum OAR doses are about the same (IMNT plans has slightly lower OAR doses than the 3DCNT plans had). Because the OAR constraints are decreased by 10% and 20%, the mean and maximum OAR doses are decreased by factors of 1.2 to 1.8 (20%-80% reduction in OAR dose). However, plans optimized with OAR tolerance doses 20% less than the corresponding ones used for 3DCNT, required a slight compromise of the PTV coverage by 3%, as quantified by the D_95%_ metric.

**Table 2.** Planning target volume (PTV) and organ at risk (OAR) doses averaged over all 7 patients. Wilcoxon signed-rank tests were used to identify significant differences (P < .05) between the intensity-modulated neutron therapy (IMNT) and 3-dimensional conformal neutron therapy (3DCNT) plans, notified with an asterisk. All values are expressed in centigray (cGy) unless otherwise noted as percentages.

**PTV and OAR averages**	**3DCNT**	**IMNT**	**IMNT_10_**	**IMNT_20_**
PTV mean, cGy	987	987	977	976
PTV D_95_, %	96	97	95	93*
PTV D_2_, %	109	111	111	113
Larynx mean, cGy	414	291*	254*	227*
Spinal cord maximum, cGy	389	327	307*	289*
Temporal lobe maximum, cGy	607	561*	541*	503*
Cerebellum maximum, cGy	433	409	382	321*
Cochlea maximum, cGy	157	127	119	100*

**Abbreviations:** IMNT_10_, reductions from the initial OAR constraints in the optimization by 10%; IMNT_20_, reductions from the initial OAR constraints in the optimization by 20%; D_95_, 95% of the prescription dose; D_2%_, near the maximum dose.

Dose-limiting OARs with average dose limitations (eg, the larynx and oral cavity) decreased in the IMNT plans for all 7 patients relative to their corresponding 3DCNT plans. The mean decrease of the average dose across patients was 43% and 49% for the larynx and oral cavity, respectively. The OARs with maximum dose limitations had decreases in their maximum dose in 21 of 25 cases when IMNT was used as the planning technique, and all decreased in maximum dose in the IMNT_20_ plans (**Table 1**). For the temporal lobe, cerebellum, and cochlea, the average maximum dose decrease was 20%, 26%, and 33%, respectively, for the IMNT plans compared with the 3DCNT plan. In 1 case (patient 2), the cerebellum and cochlea maximum doses initially increased when using IMNT but decreased as the OAR dose constraints were reduced by 20% (IMNT_20_). These maximum doses were within the tolerance limits of the plan, which implies that the optimizer allowed a higher dose in the cochlea to maintain the PTV coverage. Once the constraint was limited by the 20% reduction (ie, the maximum dose became relevant), the maximum OAR dose was lessened. Spinal cord dose was reduced by an average of 20.5% for all IMNT plans compared with the corresponding 3DCNT plan, with the maximum absolute dose, decreasing from 577 cGy to 295 cGy in patient 2, close to a 2-fold reduction in the maximum dose.

**Figure 2.**
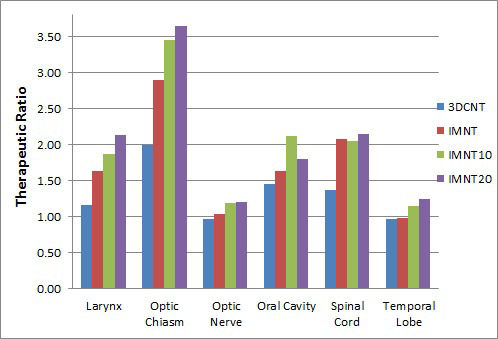
Therapeutic ratio (TR) for each organ at risk (OAR) for a representative patient case. The TR is defined as the ratio of average tumor dose to the maximum or mean OAR dose, depending on the OAR limitation. The intensity-modulated neutron therapy (IMNT)_10_ and IMNT_20_ refer to reductions from the initial OAR constraints in the optimization by 10% and 20%, respectively.

**Figure 3.**
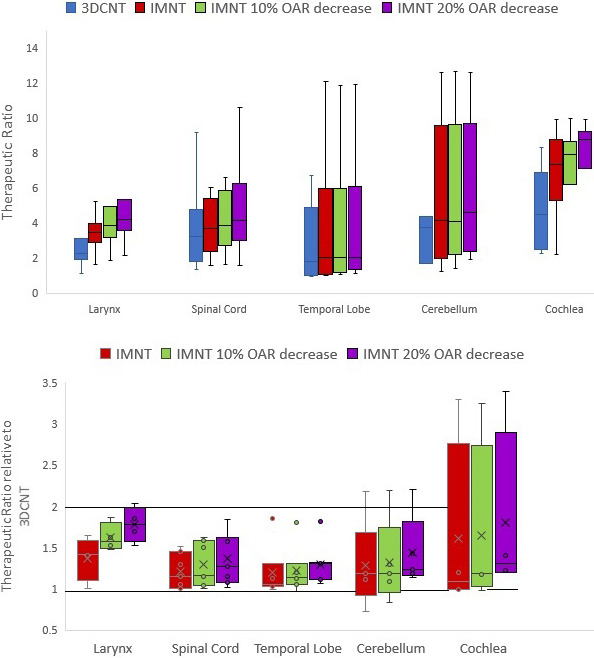
Therapeutic ratios for organs and risk (OARs) in the 7 head and neck plans. (A) The therapeutic ratio (TR) for 3-dimensional conformal neutron therapy (3DCNT), intensity-modulated neutron therapy (IMNT), and reductions from the initial OAR constraints in the optimization by 10% (IMNT_10_), and 20% (IMNT_20_), respectively. This is calculated from Equation 1. (B) The relative change of each IMNT iteration's TR. These are normalized to the 3DCNT plans. Reference lines are added at TR values of 1 and 2.

To quantify the change in OAR dose with respect to the PTV coverage, the therapeutic ratio was calculated for each OAR. **Figure 2** shows a representative case of the TR (the ratio of the mean PTV-to-OAR dose) for a representative patient with right parotid treatment (same patient as in **Figure 1**). This metric shows how, for a given tumor prescription and OAR constraint, the mean or maximum dose to the OAR changed. To show trends in how OARs changed for all patients, the TR for all 7 patients with head and neck plans is shown in **Figure 3** for representative OARs.

**Table 3** shows the mean therapeutic ratios averaged over the patients in this study. Significant increases in the TR were seen for all OARs, especially when the optimization goals were decreased to 10% and 20% of their typical values (IMNT_10_ and IMNT_20_). Only OARs contoured in ≥ 4 plans were used for analysis in **Figure 3** and in **Table 3**. **Figure 3A** shows the therapeutic ratios for 3DCNT, IMNT, IMNT_10_, and IMNT_20_. All OARs showed a systematic improvement in the TR for IMNT compared with 3DCNT. This suggests that the OAR dose decreases with IMNT relative to 3DCNT for the same mean PTV coverage.

**Table 3.** Analysis of the mean therapeutic ratio for all 7 patients with head and neck cancers. The therapeutic ratio is defined as the ratio of the mean or maximum dose to the organ at risk (OAR), depending on the OAR type. Statistically significant (P < .05) changes in the therapeutic ratio are indicated with an asterisk.

**OAR**	**3DCNT**	**IMNT**	**IMNT_10_**	**IMNT_20_**
Larynx	2.7	3.5	4.3*	4.7*
Spinal cord	3.86	4.49	4.75*	4.97*
Temporal lobe	3.04	4.17	4.18	4.27*
Cerebellum	4.43	5.91	5.99*	6.26*
Cochlea	4.89	6.72	7.00*	7.58*

**Abbreviations:** 3DCNT, 3-dimensional conformal neutron therapy; IMNT, intensity-modulated neutron therapy; IMNT_10_, reductions from the initial OAR constraints in the optimization by 10%; IMNT_20_, reductions from the initial OAR constraints in the optimization by 20%.

To compare the change in TR across all 7 patients and both treatment modalities, we normalized the TR to the TR for the corresponding 3DCNT plan. The relative TR computed in this way is shown in **Figure 3B**. A value of unity indicates no change in the TR, whereas plan quality, as judged by this metric, increases as the TR grows for IMNT plans. The TR values in the range from about 1.0 ± 0.05 are of little practical significance in light of uncertainties in patient setup and dose delivery. We consider TR values in excess of 1.2 (≥ 20%) as clinically significant because they indicate opportunities for significant tumor-dose escalation *or* a substantial decrease in potential OAR treatment toxicity. Therapeutic ratios for all OARs increased (value greater than unity in **Figure 3B**) with IMNT, with the exception of the cerebellum for patient 2, which was discussed previously. Across all OARs, the average increase in the therapeutic ratio for the IMNT_20_ plan compared with the 3DCNT plan was 49%, with a maximum increase of 239% for the cochlea in 1 plan. The average reduction in spinal cord maximum dose was 26%, with a maximum decrease of 322 cGy. This corresponded to an 84% increase in the therapeutic ratio (mean dose to PTV relative to the maximum spinal cord dose) for that plan.

## Discussion

This study retrospectively compared patient plans for conformal neutron therapy to IMNT. Reductions in OAR dose were found to be significant with comparable tumor coverage, especially in OARs near the PTV. Typical 3DCNT plans are created with a combination of 2 to 4 wedged fields. For this study, the initial treatment beam angles were used to generate the IMNT plans. As shown in **Figure 1**, IMNT shows large reductions (10%-20%) in previously wedged 3DCNT fields. The reductions in OAR dose arise because the segments in an IMNT field can close the MLCs and reduce dose in the toe of the 3DCNT wedged field for part of the delivery time. Segmented fields are also able to modulate to the treatment field in other ways that further spare OARs in close spatial proximity to (or even within) the PTV. For example, in 1 patient with oral cavity cancer (patient 4), the PTV was within 2 cm of the cerebellum. By allowing the MLCs to conform around the tumor and block the cerebellum for several segments, its maximum dose was decreased.

In addition to the dosimetric benefits, the use of IMNT and automated segment delivery to modulate the dose distributions will reduce field setup and delivery time. For example, therapists are required to enter the treatment vault to confirm beam and couch parameters before delivering a new field. Because fields with multiple segments are treated as a single field for beam-verification purposes, increase levels of dose modulation can be achieved with IMNT for the same or lower fraction delivery time than can be achieved with 3DCNT. Direct comparisons of the total delivery time for IMNT and 3DCNT treatments are yet to be established, but the total number of monitor units for 3DCNT and IMNT treatments differ by < 10% for all the reported plan comparisons, which implies that total beam on time will be about the same (within 10%) for IMNT for 3DCNT plans. For IMNT plans with the same or fewer fields than a 3DCNT treatment, the overall treatment time will be shorter for IMNT than it is for 3DCNT because the time required for therapists to enter and exit the treatment vault to verify beam and couch parameters is longer than the time to deliver each field.

The ability to provide more conformal dose distributions and greater healthy tissue sparing in neutron therapy presents new opportunities for treatment in patients with head and neck cancers. Fast neutrons demonstrate a steep dose-response curve for neurotoxicity and other late-responding tissues [38]. This has limited the use of neutron therapy in some patients and required mixed-beam approaches in others. We have commonly supplemented neutron therapy with protons or Gamma Knife stereotactic radiosurgery in patients with head and neck tumors in close proximity to, or invading the base of, the skull to overcome those limitations. The OAR constraints used in 3DCNT are defined as a compromise with current treatment limitations to maximize coverage and minimize toxicity. In properly selected patients, the OAR constraints in use at the UW cause minimal toxicity relative to historical standards [15]. The IMNT provides an exciting opportunity to push the therapeutic window for fast-neutron therapy. This may allow dose escalation to tumor volumes, where previously, tumor coverage had to be sacrificed to meet healthy tissue constraints. Improved OAR sparing may also help reduce acute and late toxicity of the neutron treatment. Reduction in mucositis, xerostomia, and dysphagia can translate into improved patient quality of life.

There are several limitations to this study and to the implementation of IMNT. This study evaluated differences in 2 neutron-delivery techniques; it did not compare neutron distributions in combination with protons or photons. Comparisons would require detailed analysis of the RBE-weighted dose distributions for the combined modality to understand the overall therapeutic benefit of IMNT relative to 3DCNT. Such considerations are beyond the scope of this work. In addition, IMNT adds new uncertainties in quality assurance and clinical throughput that will need to be evaluated. With more complex MLC patterns for delivery, quality assurance of beam delivery becomes more challenging. A neutron-sensitive pretreatment quality-assurance system is currently being verified for use before patient treatments. Although IMNT has been commissioned previously [32], our institution will be the first to implement it clinically. We expect to begin offering IMNT to selected patients with head and neck cancers in late spring of 2021. We are developing a clinical trial to evaluate the efficacy, safety, and efficiency of IMNT for salivary gland neoplasms to prospectively evaluate these concerns and collect updated data on clinical outcomes and toxicity of neutron therapy.

This study demonstrates that IMNT has the ability to significantly reduce the dose to dose-limiting OARs relative to 3DCNT plans. The increased OAR sparing available with IMNT planning is especially important for those patients who have received prior radiation therapy. As the UW CNTS is the only operating high-LET radiotherapy facility in the United States, the clinical introduction of IMNT has the potential to guide and inform the radiation oncology community on the potential benefits of high-LET radiations, including carbon ions, for the treatment of diseases that are resistant to low-LET MV x-rays and protons. The improvements reported in our study suggest that IMNT can either be used to reduce OAR toxicity or to escalate the dose to tumor. In addition to head and neck cancers, IMNT may be advantageous for many other radiation-resistant cancers, such as locally advanced prostate cancer and soft tissue, bone, and cartilage sarcomas. High-LET radiation (neutrons and carbon ions) may have a synergistic effect with immunotherapy drugs to create antitumor immune responses [17, 39, 40] that enhance the curative and palliative care for the treatment of metastatic cancers. The combination of mechanisms responsible for antitumor responses initiated by particle therapy is not yet well understood. Regardless, reducing the dose to healthy tissues with IMNT may create novel avenues to treat cancers for patients without other viable treatment options. We envision future clinical trials that provide compelling evidence that IMNT, possibly in combination with immunotherapy, provides new hope for patients with disease that cannot be effectively treated with low-LET modalities, such as photon or proton therapy.
