# Supplementary material for: Comparisons of 3-Dimensional Conformal and Intensity-Modulated Neutron Therapy for Head and Neck Cancers
Source: Int J Part Ther. 2021 Sep 14;8(2):51–61. doi: 10.14338/IJPT-20-00059.1 (PMC8489487; doi:10.14338/IJPT-20-00059.1)
Supplement: Supplementary file 1 [file ijpt-08-02-06_s01.docx]

Supplementary Table A: Patient prescription levels and OAR tolerance doses used for the clinical 3DCNT plans

| Patient # | Disease Site | Prescription (dose x n fractions) | Spinal Cord max | Larynx mean | Temporal Lobe max | Cerebellum max | Cochlea max |
| --- | --- | --- | --- | --- | --- | --- | --- |
| 1 | Parotid | 115x11 | 1100 | 800 | 1200 |  |  |
| 2 | Parotid | 115x10 | 1000 | 500 | 1250 | 1350 | 700 |
| 3 | Oral Cavity | 150x3 | 150 | 150 | 300 | 300 | 350 |
| 4 | Oral Cavity | 150x3 | 100 | 150 | 300 | 300 | 300 |
| 5 | Oral Cavity | 150x3 | 200 | 150 | 300 | 300 | 200 |
| 6 | Oral Cavity | 115x10 | 1100 |  | 1300 |  |  |
| 7 | Parotid | 200x10 | 400 |  | 1200 | 1200 |  |
